# Measurement of the Femoral Anteversion Angle in Medium and Large Dog Breeds Using Computed Tomography

**DOI:** 10.3389/fvets.2021.540406

**Published:** 2021-03-05

**Authors:** Ahmad Al Aiyan, Ken Richardson, George Manchi, Mário Ginja, Leo Brunnberg

**Affiliations:** ^1^Department of Veterinary Medicine, College of Food and Agriculture, United Arab Emirates University, Al Ain, United Arab Emirates; ^2^College of Veterinary Medicine, School of Veterinary and Life Sciences, Murdoch University, Perth, WA, Australia; ^3^Department of Veterinary Medicine, Small Animal Clinic, Freie University Berlin, Berlin, Germany; ^4^Department of Veterinary Science, Centre for the Research and Technology of Agro-Environmental and Biological Sciences (CITAB), University of Trás-os-Montes and Alto Douro, Vila Real, Portugal

**Keywords:** computed tomography, total hip replacement, canine, femoral anteversion angle, femoral morphology

## Abstract

To promote the development of an optimally functional total hip prosthesis for medium and large dog breeds, accurate measurements of the normal anatomy of the proximal femur and acetabular retroversion are essential. The aim of the current study was to obtain precise normal values of the femoral anteversion angle using computed tomography on cadavers of mature dogs with normal hip joints of both medium and large breeds. Based on the length of their femora 58 dogs were allocated either to group I: ≤195 mm or group II: >195 mm. In the study the femoral anteversion angle (FAA) was measured on each femur using multi-slice spiral computed tomography (CT). The data were processed as multi-planar and three-dimensional reconstructions using Advantage Workstation software. The CT measurements showed that the mean ± standard deviation (SD) FAA of group I was 31.34 ± 5.47° and in group II it was 31.02 ± 4.95°. There were no significant mean difference associations between the length of the femur and the femoral neck angle in either group (*P* > 0.05). The data suggest that a prosthesis FAA of 31 degrees would be suitable for a wide range of dog sizes.

## Introduction

The hind limb is frequently affected by several orthopedic diseases, such as hip dysplasia especially in medium and large dog breeds (1–6). The femoral anteversion angle (FAA) is a significant and frequently used measure for understanding the orientation of the proximal end of the femur (7–9). It plays an important role in the assessment of the health of the hip joint due to its involvement in the development of coxarthrosis in dogs (1, 10). The FAA is defined as the angle formed by the intersection of the axis of the femoral neck and the transcondylar axis of the femur, which is the axis parallel to the medial and lateral posterior edges of the condyles in the condylar plane (4). It indicates the degree of torsion of the femoral neck and head cranially and represents external rotation of the femoral neck and head relative to the distal femur (11–13). It is important biomechanically in the transfer of forces from the femur to the acetabulum (14). In a larger than normal FAA, the lever arm between the center of the femoral head and the greater trochanter is shortened (14). Thereby, the pressure, that acts on the femoral head in the acetabulum, is higher. Anatomists and surgeons have long been interested in the FAA since it is considered an important factor for hip joint stability (1, 2, 4, 8, 9, 15).

Surgical treatment of serious hip joint problems often requires total hip arthroplasty. Both the femoral neck angle and the femoral anteversion angle, that describe the relationship between the femoral head, neck and the femur shaft, must be taken into account in the development of hip endoprostheses in order to reduce the risk of hip luxation following the implantation of the prosthesis (16). Using a total hip replacement prothesis with an inappropriate FAA value may result in premature wear and loosening between the prothesis stem and the internal surface of the femoral shaft due to the increased pressure which finally cause failure of the prosthesis.

Many different methods have been used to determine the FAA, including standard radiography (1, 12, 17), biplanar planar radiography (4, 7, 18–21), computed tomography (CT) (9, 12, 22, 23), magnetic resonance imaging (24), three-dimensional modeling (25) and three-dimensional (3D) laser scanner techniques (8, 26).

Using single standard radiographic imagery to measure the FAA does not truly reflect spatial relationships between pertinent landmarks, due to a lack of depth information (27). CT imaging is considered to be a reliable and an accurate method for measuring the FAA because it allows accurate 3D volumetric femoral reconstructions of the femur and avoids artifacts due to incorrect positioning thus improving the precision of FAA measurements (20, 28–30) with average errors of 0.45° (30).

The main aim of this work was to use CT to obtain precise data of the femoral anteversion angle in cadavers of medium and large dog breeds in support of the development of an optimally functioning total hip replacement prothesis. In addition we provide a detailed description of the methodology using CT to measure the femoral anteversion angle.

## Materials and Methods

The cadavers used in this study have been reported earlier in a previous article where the femoral neck inclination angle was studied (31).

Femora from 58 cadavers of orthopedically healthy adult dogs of medium and large breed size were studied using computed tomography. The dogs used in this study were obtained from the Small Animal Clinic of the Free University of Berlin. The dogs had either died or were euthanased for reasons unrelated to this study. For each individual dog the research ethics code of the institution was met and accompanied by written consent from the dog's owner.

Post-mortem examination was conducted on each dog to establish the absence of orthopedic abnormalities and disease. The Ortolani and Barlow tests were conducted immediately post-mortem. Radiography and CT examination of the hip joint was conducted post-mortem immediately after the death to establish the absence of hip joint dysplasia. The dogs used in this study had no clinical history of pelvic limb lameness. Dogs with orthopedic abnormalities or signs of hip joint disease were excluded from the study.

The dogs used in this study were assigned into two groups according to the length of their femora measured in CT (32).

The CT scanning was conducted at the Small Animal Clinic, Düppell, Free University of Berlin. The CT scanning of the femora was done at a setting of 0.3 mm slice thickness, multi-slice spiral “Lightspeeds” QXi (General Electric Healthcare, GE), 120 kV, 130 mAs. The dogs were positioned in dorsal recumbency on the CT scanner table. The pelvic limbs were pulled back and tied at the tarsal level with adhesive straps (Tesa AG Humburg) to ensure that the femora were parallel to each other and parallel to the CT scanner table. Advantage Workstation software (Advantage Workstation 4.2, GE Healthcare) was used to analyse the images. The data record was processed as multi-planar and three-dimensional reconstructions using Advantage Workstation software.

The sequence of measurements were done in the following order as some measurements were reliant on values of earlier measurements: determination of the axis of the femoral shaft, length of the femur, center of the femoral head, axis of the femoral neck, condylar axis, femoral anteversion angle. All measurements were performed by an experienced veterinarian and repeated after 24 h. The mean of the two measurements of the FAA was used to ensure data accuracy.

### Medullary Axis and Length of the Femur

**Figure 1:**
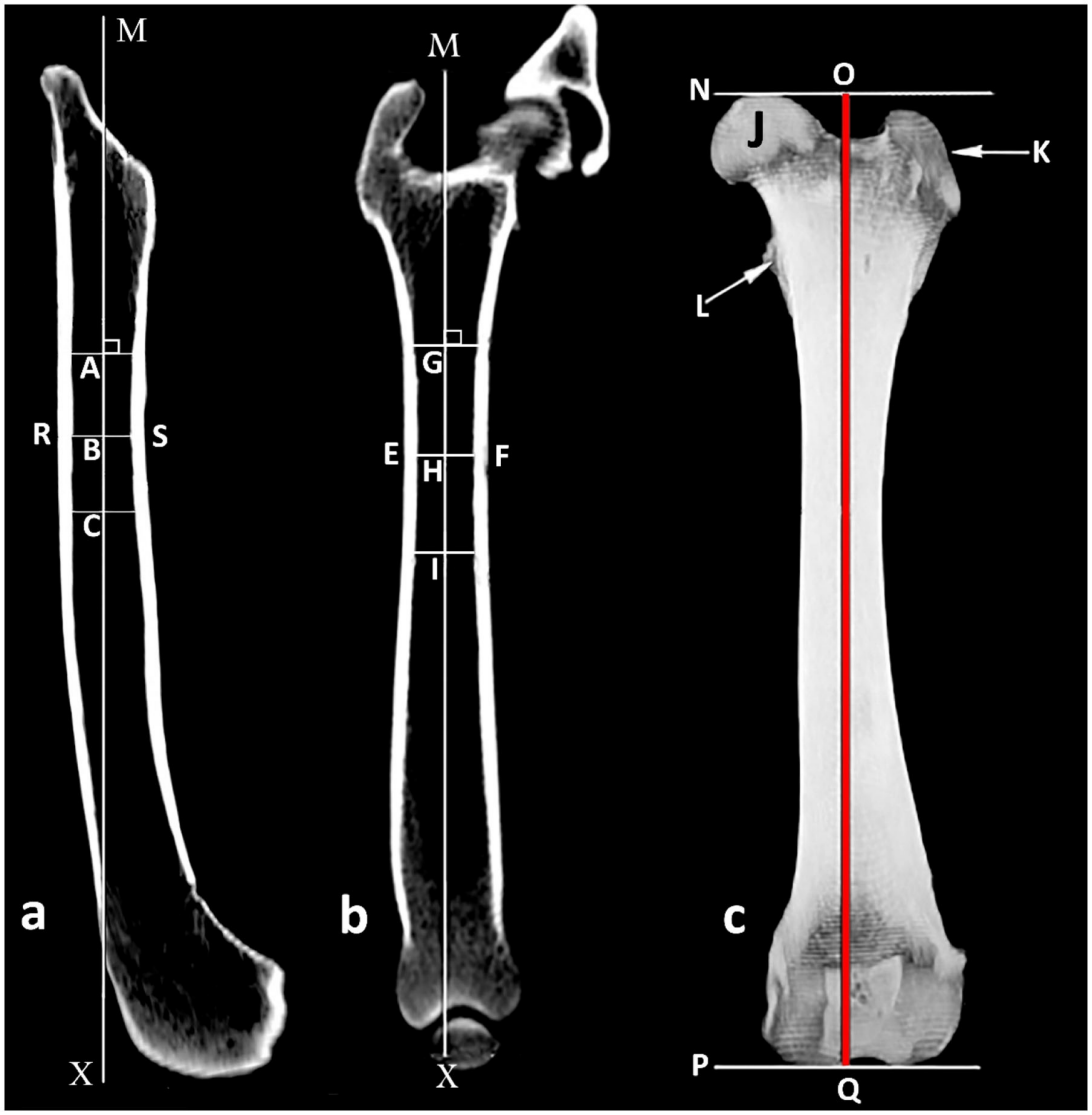
Determination of the medullary axis of the femoral shaft. **(a)** Sagittal plane view of the femur where line RS represents the intracortical width at the narrowest point of the femoral shaft and B is its central point; A and C are central points 2 cm proximal and 2 cm distal to (B) respectively; MX is the medullary sagittal axis. **(b)** Frontal plane view of the femur where EF represents the intracortical width at the narrowest point of the femoral shaft and H is its central point; G and I are central points 2 cm proximal and 2 cm distal to H, respectively; MX is the medullary frontal axis. **(c)** Frontal view of the femur where N and P are the proximal and distal orthogonal lines to medullary axis, respectively; J femoral head; K great trochanter; L lesser trochanter; OQ length of the femur.

To ensure consistency in femoral measurements, an exact sagittal plane view was obtained by aligning the caudal aspects of both femoral condyles, and to avoid cranial or caudal inclination of the femur, the femoral axis was identified as the line connecting the three central points shown in Figure 1a and was placed vertically (Figure 1a). From here the femur was rotated exactly 90° cranially to be able to obtain an accurate frontal plane view of the femur without external or internal rotation (Figure 1b).

In the sagittal and frontal planes, the center of the intracortical width was created at the narrowest point of the femoral shaft. Using similar methodology, additional central points were placed 2 cm proximal and 2 cm distal. The axis of the femoral shaft was identified as the line connecting the three central points (Figures 1a,b). Using a three dimensional model in a frontal view, the length of the femur was determined to be the line parallel to the femoral axis that connects the orthogonal lines at the most proximal point of the femoral head and at the most distal end of the femoral condyles (Figure 1c).

### Center of the Femoral Head

**Figure 2:**
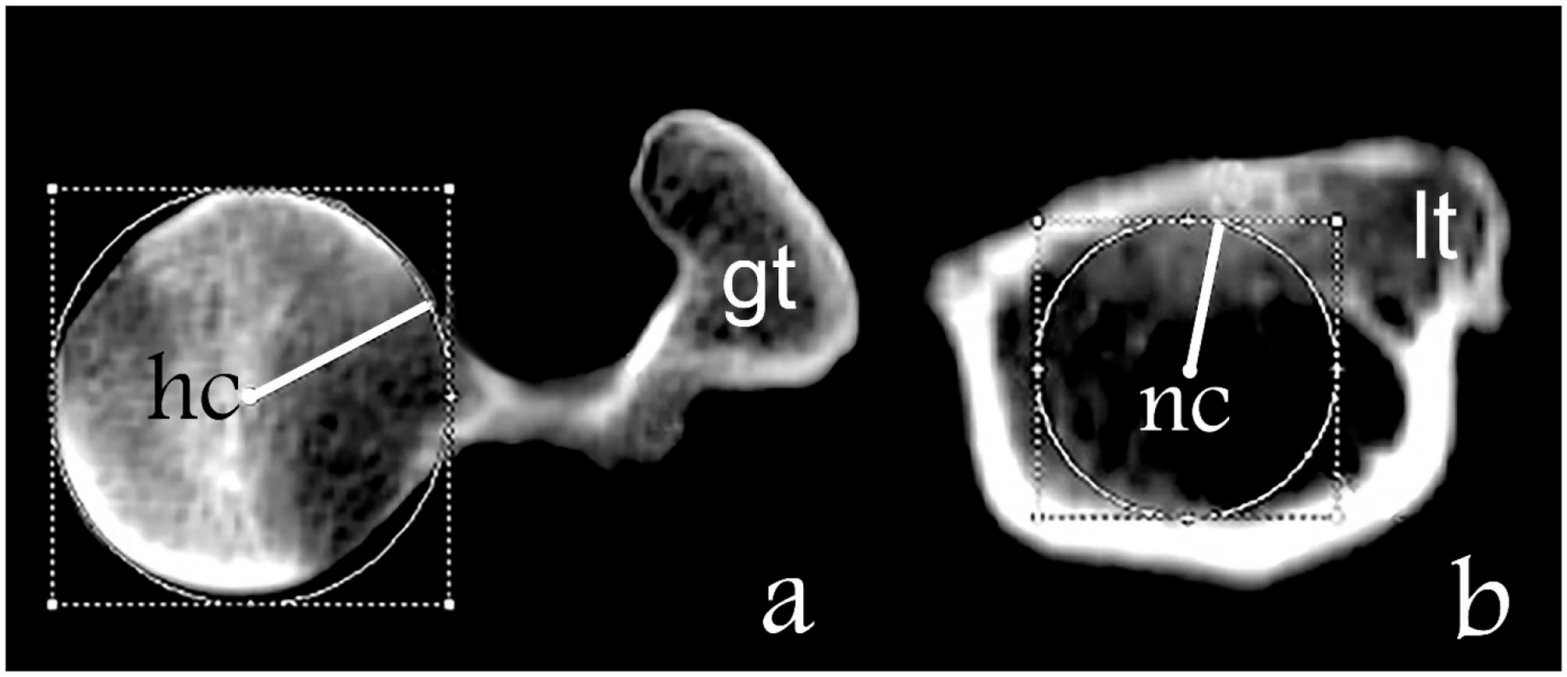
Determination of the axis of the femoral neck. **(a)** Transverse plane of the proximal femur where “gt” is the great trochanter and “hc” is the center of the femoral head. **(b)** Transverse plane of the proximal femur on the level of the lesser trochanter where “lt” is the lesser trochanter and “nc” is the center of the base of the femur neck.

Using a 3D transverse plane, the center of the femoral head was identified by using annotation software to generate concentric circles of best fit and superimpose these onto the femoral head (Figure 2a).

### Axis of the Femoral Neck and the Condylar Axis

**Figure 3:**
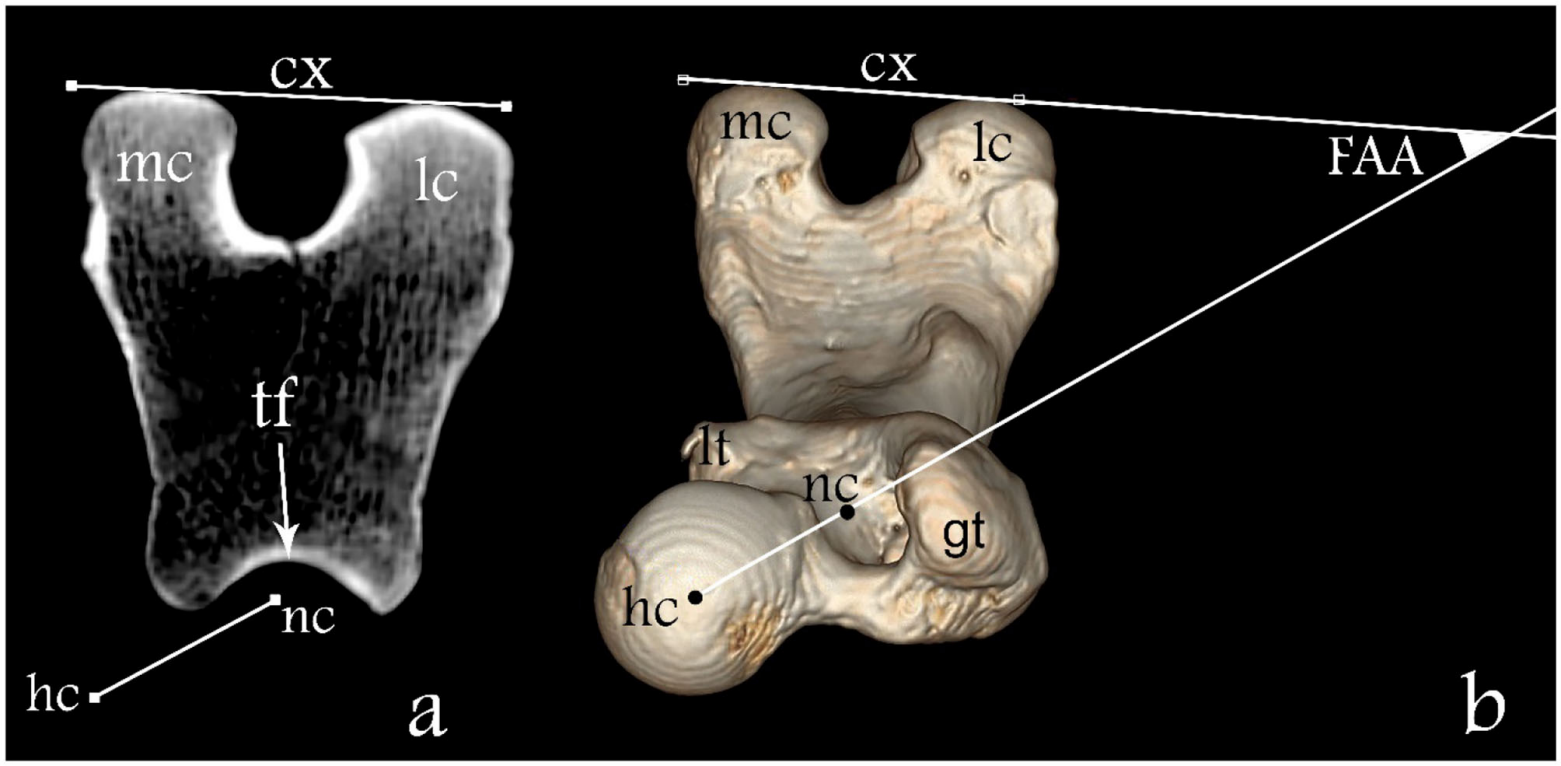
Determination of the FAA. **(a)** Transverse plane overlap view of the distal femur and **(b)** three-dimensional dorsoventral view of the femur. Here “hc” represents the femoral head center, “nc” the femoral neck center, “tf” the femoral trochlea, “mc” medial condyle, “lc” lateral condyle, “gt” greater trochanter, “lt” lesser trochanter; line between “nc” and “hc” femoral neck axis and line “cx” condylar axis. The FAA angle is defined between lines “nc-hc” and cx.

In the transverse femoral neck planes the lesser trochanter appears at the transition from the medial to the caudal contour of the femur and disappears in more distal sections. A computer-generated circle was placed in the section with the maximum extent of the lesser trochanter and the center of the circle was determined and presents the base of the femoral neck (Figure 2b). The axis of the femoral neck was defined as the line passing from the center of the femoral head to the base of the femoral neck in the transverse view of the femur and remained visible on the monitor (Figures 2a, 3a). More distally, the sectional view with the maximum caudal curvature of the condyles was defined to represent the condylar axis (Figure 3a). The FAA was measured between the femoral neck axis and the condylar axis (Figure 3b).

### Statistical Analysis

The statistical analysis was based in the comparison of FAA in the two groups with different femoral length. The intra-class correlation coefficient (ICC) and Kendall's tau was used in order to evaluate the intra-observer independent measurement repeatability. The Pearson correlation was used to study the association between the length of the femur and the femoral anteversion angle. Pearson and Kendall's tau results of −1 or 1 indicate perfect negative or positive association between variables. A *P*-value smaller than 0.05 was considered significant. The statistical analysis was performed using the Statistical Packages for Social Science software (SPSS Inc. Version 26, Chicago II, USA). Values were reported as mean +/– standard deviation.

## Results

In this study a total of 116 femora were measured from 58 medium to large breed dogs. Twenty-three dogs were excluded from the study due to orthopedic abnormalities or signs of hip joint disease which had been detected. The most common breed measured was the German Shepherd followed by Staffordshire Terriers, Boxers, Rottweilers, Bullmastiffs, and Weimaraners.

Dogs were divided into two groups according to the length of their femora. Group I (*n* = 25) included all the dogs with a femoral length ≤195 mm. Group II (*n* = 33) included all the dogs with a femoral length >195 mm.

The age of dogs in Group I ranged from 2 to 16 years old, mean 7.6 ± 4.15 years and in Group II ranged from 1.5 to 16 years old, mean 8.4 ± 3.95 years. The body mass of dogs in Group I ranged from 17 to 45 kg, mean 27.8 ± 7.53 kg, and in Group II ranged from 22 to 60 kg, mean 42.3 ± 8.37 kg.

All the measurements performed in the two independent sessions had adequate repeatability as the Kendall's tau test showed strong correlation (tau = 0.956, *P* = 0.000). The femur length in Group I was 175.29 ± 12.29 mm and in Group II 213.44 ± 15.77 mm.

**Figure 4:**
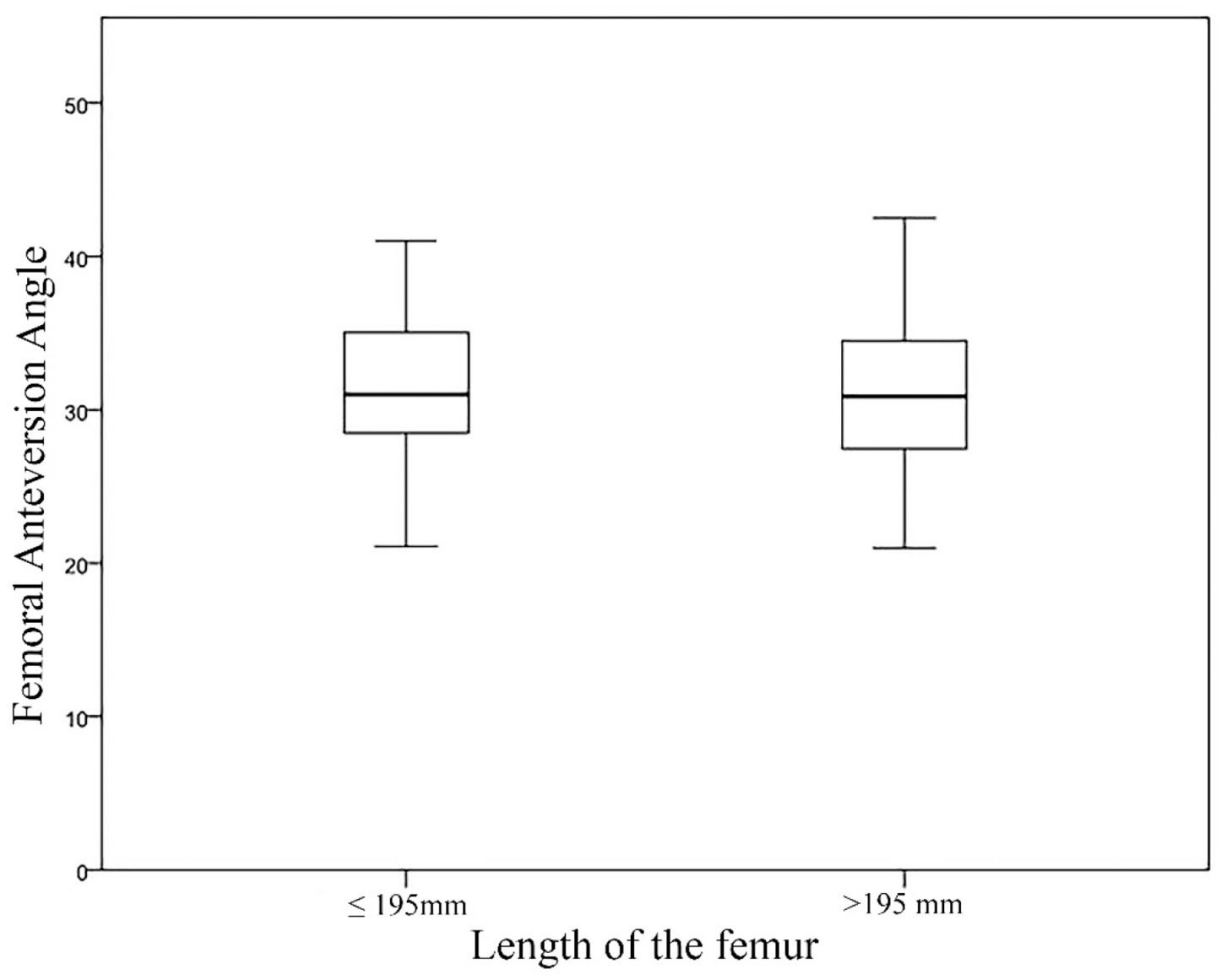
Boxplot with medians and data ranges representing the Femoral Anteversion Angle (FAA) in relation to the Length of the Femur for Group I and Group II.

The mean values of FAA obtained in this study were 30.99 ± 4.02° for Group I and 31.58 ± 5.09° in Group II. No correlation was found between the length of the femur and the FAA (*P* = 0.136) (Figure 4).

## Discussion

Because the dogs used in this study varied in their nutritional status and history, body mass was not included as a morphological parameter (32). The medium to large breed dogs used in the present study were assigned to two groups based solely on the total length of their femur (32). The FAA measured in this study shows no significant difference between group I and group II. Likewise, there was no correlation between the length of the femur and the FAA (Figure 4). This is consistent with the results of Palierne et al. (32).

In adult medium and large dog breeds with normal hip joint morphology, the FAA has been measured using several different imaging methods as well as anatomical preparation and reported in the literature to be within the range of 7.6 to 34.2° (3, 4, 7–9, 12, 23, 24, 33–35). The results reported vary greatly in these studies (Table 1). However, there are many relatively common congenital and developmental conditions where the FAA deviates significantly from the normal such as canine hip dysplasia associated with a larger than normal FAA, that tends to rotate the femoral head out of the acetabulum (1, 2). The different measurement methodologies as well as the body size, age profile, gender, and breeds of dog populations, may explain the different results (15, 27).

**Table 1:** Mean (SD) femoral anteversion angle reported in dogs by other studies, measured by standard radiograph (RAD), computed tomography (CT), magnetic resonance imaging (MRI) and anatomical preparation (AP).

**Authors**	**N**	**Method**	**FAA (SD)**
Adams et al. (8)	five mongrel dogs	3D scanner and 3D animation software	23.4° ± 3.5
Bardet et al. (19)	15 mixed, medium to large	Fluoroscopic method	31.31°
Bardet et al. (19)	15 mixed, medium to large	RAD biplanar	30.8°
Bloebaum et al. (34)	21 greyhound	RAD biplanar	27° ± 6.3
Dudley et al. (12)	nine medium to large	RAD, Fluoroscopic method	16° ± 6.4
Dudley et al. (12)	nine medium to large	CT	19.6° ± 7.9
Dudley et al. (12)	nine medium to large	AP	18.9° ± 5.4
Ginja et al. (23)	23 estrela Mountain Dogs, 7–8 week	RAD biplanar	29.9° ± 4.8
Ginja et al. (23)	23 estrela Mountain Dogs, 7–8 week	CT	30.4° ± 4.2
Griffon et al. (7)	160 labrador Retrievers	RAD biplanar	29.67° ± 6.44
Hauptman et al. (3)	75 medium to large	RAD biplanar	15.2°
Kaiser et al. (24)	40 small, medium to large	MRI	7.6° ± 5.5
Kara et al. (9)	75 mixed breeds	CT	26.86° ± 11.46
Löer (36)	large breeds	CT	33.8°
Löer (36)	small breeds	CT	33.2°
Madsen and Svalastoga (37)	41 medium to large	RAD biplanar	30°-43°
Mahringer, (38)	105 medium to large	AP	33° ± 8.66
Montavon et al. (4)	30 mongrel dogs, medium to large	RAD biplanar	31.3° ± 6.2
Martins et al. (21)	126 young normal joints	RAD biplanar	31.4° ± 4.8
	106 young abnormal joints		32.6° ± 4.9
	158 adult normal joints		26.4° ± 4.5
	232 adult abnormal joints		27.7° ± 5.0
Nunamaker et al. (1)	34 various breeds adults	RAD, Fluoroscopic method	26.97° ± 6.52
Palierne et al. (35)	82 medium to large	RAD biplanar	30° ± 6.32
Palierne et al. (32)	206 small, medium to large	RAD biplanar	29.40° ± 6.35
Savio et al. (26)	16 medium to large	3D scanner and design software	45° ± 4.5
Schawalder et al. (11)	50 medium to large	RAD biplanar	30.1°
Sumner et al. (33)	15 medium to large	RAD biplanar	34.2° ± 5.7

Accurate measurement of the FAA using classical radiography relies on precise positioning of the femur to obtain a true axial projection of the femur from distal to proximal, which is technically challenging due to the difficulties encountered in patient positioning (12). Often multiple attempts are necessary; consequently such radiographic studies can often be time-consuming (12).

Due to the complex three-dimensional configuration of the femur, CT imaging is considered to be the most reliable and accurate method to measure the FAA (9, 12, 20, 26, 28, 29). This allows accurate 3D volumetric femoral reconstructions of the femur and obviates artifacts related to animal position and thereby increases the precision of the FAA measurement (12, 20, 28, 29) and can be used for clinical or research purposes without the need of additional radiographic exposures (12, 23).

The patient preparation and the time required for radiographic and CT examinations are similar (23). Even using the same imaging technique could result in different values, due to the different methodologies used to estimate the center of the base of the femoral neck (27). Minor variations in radiographic positioning and selection of landmarks affect the correctness and variability of radiographic measurements (13).

In the present study, the precise FAA was obtained using a CT scan data set of 116 femora of 58 mature dogs, all free of hip dysplasia. Multi-slice spiral computed tomography and Advantage Workstation software were used for the analysis. A set of five landmarks; the center of the femoral head, center of the base of the femoral neck, lesser trochanter, medial and lateral aspect of the femoral condyles were found to be readily identifiable and suitable for our CT measurements.

In this study the mean value of the FAA in dogs with a femoral length of between 145 and 195 mm (group I) is 30.99 ± 4.02° and in dogs with a femoral length of between 196 and 240 mm (group II) is 31.58 ± 5.09°. The mean FAA reported in the present study are in close agreement with those of Schawalder and Sterchi (11), Bardet et al. (19), Montavon et al. (4), Sumner et al. (33, 38)), Löer (36), Palierne et al. (35), Ginja et al. (23), Palierne et al. (32), and (7) (Table 1).

Our findings are inconsistent with (1, 3, 12, 24, 26, 34, 37) (Table 1). The use of different measurement techniques can explain the different results of the FAA values. In the current study we found that accurate identification of the sagittal and frontal planes as demonstrated in this study are necessary to delineate the intramedullary axis of the femur. The transverse plane is the appropriate plane to identify the center of the femoral head, the femoral neck axis and the condylar axis to be able to measure the FAA. In addition the size, age, gender, and breed of the dog population also contribute to variations in the FAA (4, 11, 15, 19, 24, 25, 27, 36).

Martins et al. (21) described a significant reduction in FAA in adult animals compared to younger dogs. In contrast, the mean FAA of 7.6° in the Kaiser et al. (24) study, during which magnetic resonance imaging (MRI) was used, is considerably lower than the mean FAA seen in other studies, this could be due to fact that the femoral head center lies cranially to the plane in which we can define the center of the femoral neck (23, 24).

Some authors confirm a link between an increased anteversion angle and the incidence of degenerative hip diseases such as hip joint dysplasia (1, 2, 4) whilst some others do not (21). This could confirm the high FAA measured by Savio et al. (26) (45°) and by Madsen and Svalastoga (37) (30–43°).

The FAA can support the development of a durable and optimally functional hip prosthesis. The use of correctly designed hip prostheses plays an active role in lowering the risk of postsurgical complications associated with hip arthroplasty in medium and large dog breeds. According to this study, using the methodology described, the measurement of the FAA can be made with good repeatability by a single observer based on using femoral length as a proxy for dog size, a prosthesis FAA of 31 degrees would be suitable for a wide range of dog sizes.

## Data Availability Statement

The datasets generated for this study are available on request to the corresponding author.

## Ethics Statement

Ethical review and approval was not required for this animal study as cadavers were obtained from euthanized animals. All research activities were done in accordance with The Central Ethics Committee of Freie Universität Berlin. Written informed consent was obtained from the animal's owner for their use in this study.

## Author Contributions

All authors have contributed to the conception, design, acquisition of data, analysis and interpretation of data, drafting or revising, and final approval of the manuscript.

## Conflict of Interest

The authors declare that the research was conducted in the absence of any commercial or financial relationships that could be construed as a potential conflict of interest.
